# Population genetic diversity and dynamics of the honey bee brood pathogen *Melissococcus plutonius* in a region with high prevalence

**DOI:** 10.1016/j.jip.2022.107867

**Published:** 2023-02

**Authors:** Daniela Grossar, Edward Haynes, Giles E. Budge, Melanie Parejo, Laurent Gauthier, Jean-Daniel Charrière, Michel Chapuisat, Vincent Dietemann

**Affiliations:** a Swiss Bee Research Center, Agroscope, Schwarzenburgstrasse 161, 3003 Bern, Switzerland; b Department of Ecology and Evolution, Biophore, UNIL-Sorge, University of Lausanne, 1015 Lausanne, Switzerland; c Bee health laboratory, Fera Science Ltd, Sand Hutton, York, YO41 1LZ, the United Kingdom of Great Britain and Northern Ireland; d Fera Department of Biology, University of York, York, the United Kingdom of Great Britain and Northern Ireland; e School of Natural and Environmental Sciences, Newcastle University, Newcastle upon Tyne, Tyne and Wear NE1 7RU, the United Kingdom of Great Britain and Northern Ireland; f Applied Genomics and Bioinformatics, University of the Basque Country (UPV/EHU), Leioa, Bilbao, 48940, Spain

**Keywords:** European foulbrood, MLST, *mtxA*, Brood disease, Bacterial disease, *Melissococcus plutonius*, *Apis mellifera*

## Abstract

European foulbrood (EFB) is a honey bee brood disease caused by the bacterium *Melissococcus plutonius*. Large-scale EFB outbreaks have been reported in several countries in recent decades, which entail costly sanitation measures of affected apiaries to restrict the spread of this contagious pathogen. To mitigate its impact, a better understanding of the population dynamics of the etiological agent is required. We here used multi-locus sequence typing (MLST) to infer the genetic diversity and geographical distribution of 160 *M. plutonius* isolates collected from EFB symptomatic honey bee colonies seven years apart. Isolates belonged to three clonal complexes (CCs) known worldwide and to 12 sequence types (STs), of which five were novel. Phylogenetic and clustering analyses showed that some of these novel sequence types have likely evolved locally during a period of outbreak, but most disappeared again. We further screened the isolates for melissotoxin A (*mtxA*), a putative virulence gene. The prevalence of STs in which *mtxA* was frequent increased over time, suggesting that this gene promotes spread. Despite the increased frequency of this gene in the population, the total number of cases decreased, which could be due to stricter control measures implemented before the second sampling period. Our results provide a better understanding of *M. plutonius* population dynamics and help identify knowledge gaps that limit efficient control of this emerging disease.

## Highlights

• MLST and spatial analysis of the honey bee pathogen *M. plutonius.*
• Identification of five novel *M. plutonius* sequence types.
• Temporal increase in the occurrence of the putative virulence factor *melissotoxin A.*

## Graphical abstract

**Figure:**
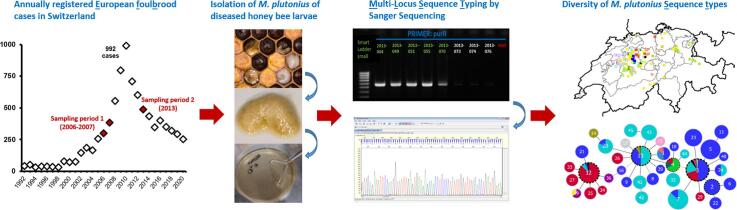

## 1 Introduction

The Western honey bee (*Apis mellifera* L.) is exploited for their natural products since at least the Neolithic era (Roffet-Salque et al., 2015), and more recently, to provide pollination service to many wild plants and economically important crops (Kleijn et al., 2015). Consequently, *A. mellifera* is one of the most commonly managed insects in the world. The large-scale losses of managed colonies reported in recent times in the Northern Hemisphere potentially threaten the services honey bees provide (Decourtye et al., 2019, Gray, 2022, Kulhanek et al., 2017, Liu et al., 2016, Neumann and Carreck, 2010, Potts et al., 2010, Steinhauer et al., 2018). Honey bee health has thus become a major concern for scientists, policy-makers, beekeepers and the public (Evans and Schwarz, 2011, Moritz et al., 2010, Smith et al., 2013). Honey bee colony losses are primarily attributed to pests and pathogens such as viruses (Berthoud et al., 2010, Genersch, 2010, Highfield et al., 2009), protozoa (Morimoto et al., 2013, Ravoet et al., 2013), fungi (Higes et al., 2008, Paxton, 2010), parasitic mites (Dahle, 2010, Dainat et al., 2012, Guzmán-Novoa et al., 2010, Kielmanowicz et al., 2015, McMullan and Brown, 2009) and bacteria (Cornman et al., 2012, Evans and Schwarz, 2011, Genersch, 2010, Roetschi et al., 2008, Wilkins et al., 2007).

One of the major bacterial diseases affecting the honey bee is European foulbrood (EFB). EFB is present worldwide (Boncristiani et al., 2020, Ellis and Munn, 2005) and has become a problem in numerous countries that have reported severe outbreaks (de León-Door et al., 2018, Grossar et al., 2020, Nesvorna et al., 2020, Pietropaoli et al., 2022, Roetschi et al., 2008, Thebeau et al., 2022, Wilkins et al., 2007). Adult honey bees act as carriers of the bacterial pathogen *M. plutonius*, but typically show no symptoms, while immature honey bees (brood) often die upon infection (Belloy et al., 2007, Forsgren, 2010). The massive loss of brood due to the disease weakens the colony and can lead to its collapse (Forsgren et al., 2013). To prevent the further spread of the bacteria, in case of outbreaks, the authorities of many countries require the destruction of affected colonies and occasionally of whole apiaries, and the implementation of costly sanitation measures, including the establishment of control zones around affected apiaries and recurrent inspections of neighboring apiaries (Forsgren, 2010).

Despite the application of strict control measures since 2010, immediately after a rise in outbreaks frequency, the situation in Switzerland has improved, but the situation is still not under control. This is in part due to a lack of effective preventive or control methods. The development of such methods is limited by our poor knowledge of the epidemiological processes that underlie the expression and spread of this disease. A better understanding of transmission routes, and of biotic and abiotic risk factors for disease outbreaks can be achieved by determining the structure, genetic diversity and dynamics of *M. plutonius* populations, as well as the phenotypic traits associated with the virulence and ability to spread of genetic variants. Knowing the genetic landscape of *M. plutonius* variants and their effects on honey bees could also help monitoring the emergence of new problematic variants, and possibly prevent their negative impacts on honey bee stocks.

In Switzerland, the frequency of EFB symptomatic apiaries rose from 1999 to peak in 2010, with 992 reported cases (InfoSM, Federal Food Safety and Veterinary Office FSVO), which corresponds to approximately 5 % of the Swiss active apiaries at this time (Charrière et al., 2018). The prevalence of EFB shows striking spatial differences in the country. The disease is more frequently detected in the central midland and eastern parts than in the western or southern parts of the country. Strikingly, the canton of Bern, which holds 23 % of the Swiss active apiaries, reported as many as 41 % of all registered EFB cases in Switzerland (N = 8596 cases 1992–2021; InfoSM, Federal Food Safety and Veterinary Office FSVO; von Büren et al. (2019)). This pattern is unexplained and might be due to multifactorial interactions between topographical and climatic differences; to anthropological influences (e.g., apiary density von Büren et al. (2019), beekeeping practices or exchange of materials within language groups); and/or to regionally specific strains of *M. plutonius*. Also, newly emerging variants of *M. plutonius* could contribute to higher local prevalence and impact of the pathogen.

In the present study, we aimed to obtain a better understanding of the factors influencing the regional distribution of *M. plutonius* outbreak by investigating the genetic diversity, population structure and dynamics of this pathogen. For this, we applied a multi-locus sequence typing (MLST) scheme to 160 isolates collected from EFB-diseased colonies across Switzerland, sampled over two periods seven years apart. MLST is a robust method to distinguish between genetic subtypes of clonal bacterial species (Killgore et al., 2008), and has been used to unravel the epidemiology of numerous human and animal pathogens (Dingle et al., 2002, Freitas et al., 2009, Lowder et al., 2009, Ruiz-Garbajosa et al., 2006). For *M. plutonius*, a MLST-scheme incorporating four genomic loci has been developed (Haynes et al., 2013) to classify *M. plutonius* strains into sequence types, themselves grouped into clonal complexes (CCs), of which three have been described to date (CC 3, CC 12 and CC 13).

Although genotyping is important to identify cases or outbreaks and to track the source and spread of infections, it is currently unclear whether the markers used reflect phenotypic variation relevant to disease progression, such as virulence (Freitas et al., 2009, Lowder et al., 2009). This is also the case for *M. plutonius*. The study of correlations between sequencing types and virulence factors could hence help to better predict EFB outbreaks and spread of the disease in the field. For instance, such correlation was shown for *M. plutonius* strains from CC 3, which exhibited stronger resistance to the antimicrobial activity of royal jelly, and could explain the higher virulence of this clonal complex (Takamatsu et al., 2017). In contrast, isolate virulence at the individual level was not related to its sequence type, but was correlated to the occurrence of a mobile-genetic element, plasmid pMP19, which carries the *mtxA* gene encoding the protein melissotoxin A (Djukic et al., 2018, Grossar et al., 2020, Nakamura et al., 2020). Because of its putative role in virulence, this gene could affect the ability of an isolate harboring it to cause an outbreak. We therefore screened the collected isolates for the presence of *mtxA.* Our results increase knowledge of *M. plutonius* genetic diversity, population dynamics, and distribution in a country with high disease prevalence. Such data on *M. plutonius* help explain differences in the spread and impact of EFB between regions, and could lead to the development of refined mitigation measures.

## 2 Methods

### 2.1 Sampling and isolation of *M. plutonius*

Routine inspections of Swiss apiaries as well as controls following the announcement of suspect cases by beekeepers are conducted by trained bee inspectors, as part of a national control program (Massnahmen im Seuchenfall von Sauerbrut (Europäische Faulbrut) bei Bienen, 10.08.2015 based on Art. 273a TSV 27. 06. 1995; SR 916.401; (FSVO) (2015)). Such inspections can entail the sampling of brood combs from colonies suspected to show EFB symptoms. These samples are sent to one of three accredited laboratories (Laboratoire vétérinaire Institut Galli-Valerio, Lausanne; IDEXX Diavet AG, Bäch; Zentrum für Labormedizin, St.Gallen) for diagnostic disease confirmation via microscopical analysis and/or PCR (Roetschi et al., 2008). Brood combs that contained larvae diagnosed positive for *M. plutonius* were obtained from these laboratories for MLST typing. To isolate *M. plutonius*, we collected 1–3 symptomatic larvae (yellowish-brown color, unpleasant smell and signs of decay) from one brood comb per infected apiary and ground them in sterile saline solution (0.9 % NaCl w/V). A droplet of 25 µl of each larval homogenate was then streaked on basal medium plates, containing 1 % yeast extract, 1 % glucose, 1 % saccharose, 0.04 % l-cysteine and 0.1 M KH_2_PO_4_ in distilled water, pH adjusted to 6.7, and 18 g agar/l, and incubated at 36 ± 1 °C for 4 to 5 days under anaerobic conditions (GENbox anaer, bioMérieux, France) (Forsgren et al., 2013). Individual bacterial colonies suspected to be *M. plutonius* were picked from the plates with sterile plastic needles and dipped into the *M. plutonius*-specific PCR mix, to confirm the bacterial species. This mix contained the primers Mp-SodA-F (5′-ACTGAAACAATGCATTTGCACC-3′) and Mp-SodA-R (5′-AGTGGTGAATCTTGGTTGGCT-3′) that target the sodA gene (Manganese dependent superoxide dismutase) of *M. plutonius* (Roetschi et al., 2008). Primers Mp-SodA-F and Mp-SodA-R were designed for this study using the tool Primer BLAST at NCBI (https://www.ncbi.nlm.nih.gov/; Ye et al., 2012). The protocol was adapted for conventional PCR, instead of RT-PCR, and the PCR amplification size was 401 bp. PCR positive subcultures were then again inoculated into liquid basal medium (1 % yeast extract, 1 % glucose, 1 % saccharose, 0.04 % l-cysteine and 0.1 M KH_2_PO_4_ in distilled water, pH adjusted to 6.7 with 5 M KOH) (Forsgren et al., 2013) and incubated anaerobically at 36 ± 1 °C for 4 to 5 d, supplemented with 15 % Glycerol and stored at −80 °C until further use.

Our dataset comprised 160 *M. plutonius* isolates from EFB symptomatic colonies. We sequence-typed 91 field isolates originating from EFB outbreaks that occurred between 2006 and 2007, and 63 originating from EFB outbreaks in 2013. The single *M. plutonius* isolate from each infected apiary was used for further analysis, except for four EFB outbreaks in 2006–2007 from which two isolates were available and analyzed. To complete the dataset, published MLST-data of four Swiss isolates collected in 2006 (http://pubmlst.org/mplutonius) were included in the analyses. The total number of EFB cases analyzed corresponded to approximately 14 % and 13 % of all reported EFB cases in 2006–2007 and 2013, respectively. In Switzerland, EFB case numbers reached a peak in 2010 (InfoSM, Federal Food Safety and Veterinary Office FSVO). Therefore, the first sampling (2006–2007) occurred during a period of increasing EFB case numbers, while the second sampling (2013) occurred during a period of decrease in numbers of notified EFB cases.

### 2.2 Genetic typing and phylogenetic analysis of *M. plutonius* isolates

DNA was extracted from single step *M. plutonius* cultures grown from isolate stock solutions in liquid basal medium, using the NucleoSpin® Tissue DNA extraction kit (Macherey-Nagel, Germany). The DNA samples were stored at −20 °C until further use. For multi-locus sequence typing (MLST) of *M. plutonius*, we PCR-amplified and sequenced the following four loci: argE (Acetylornithine deacetylase, 503 bp; argE-for: 5′-GGTGGGACATTTAGACGTAG-3′ and argE-rev: 5′-AAATTAAGACCCAACCCTTC-3′), galK (Galactokinase, 471 bp; galK-for: 5′-TTTCCAGCAGCAATTACAA-3′ and galK-rev: 5′-GGGTAGGGATTTTTGAAGAG-3′), gbpB (Putative secreted antigene, 314–557 bp; gbpB-for: 5′-AGCAGCTAAACAGAATGAGC-3′ and gbpB-rev: 5′-GCCAACGTCTAACAGATACC-3′) and purR (Purine operon repressor, 382 bp; purR-for: 5′-GCCAACGTCTAACAGATACC-3′ and purR-rev: 5′-CGATTTTGTTCTGATAACCTG-3′) (Haynes et al., 2013). For each locus, we used the KAPA2G fast Polymerase (Kapa Biosystems, USA) and a PCR mix consisting of 13 µl Kapa (2x) mix, 1 µl forward primer at 10 µM, 1 µl reverse primer at 10 µM, 8 µl water and 2 µl template DNA (approx. 10 ng DNA) in elution buffer (5 mM Tris/HCl, pH 8.5; total reaction volume 25 µl). Each reaction was run for 15 min at 95 °C, followed by 40 cycles of denaturation at 95 °C for 15 s, annealing at 58 °C for 15 s and DNA extension at 72 °C for 20 s, and a final extension step at 72 °C for 2 min in a TProfessional Basic Thermocycler (Biometra, Germany). PCR products were loaded on agarose gels (1.5 % agarose in 0.5 % TBE buffer (45 mM Tris-borate, 1 mM EDTA, pH 8)), run for 40 min at 100 mV in an electrophoresis tank and stained for 20 min in a GelRed™ bath (Biotium, USA). Bands visible under UV-light were sized according to a DNA ladder (SmartLadder SF, 100 to 1,000 bp molecular weight marker; Eurogentec, Belgium). PCR products of the expected size were purified with the NucleoSpin® Gel and PCR Clean-up kit (Macherey-Nagel, Germany). The DNA content of purified extracts dissolved in elution buffer (5 mM Tris/HCl, pH 8.5) was measured using NanoDrop™ (Thermoscientific, USA). Extracts were diluted, supplemented with the corresponding primer, and sent for Sanger sequencing (Eurofins Scientific, Germany or Fasteris SA, Switzerland). Sequencing chromatograms were manually checked for their quality using Sequence Scanner v1.0 (Applied Biosystems, 2005) and aligned to reference sequences from the PubMLST database (http://pubmlst.org/mplutonius, as per June 2, 2022; Bacterial Isolate Genome Sequence database (BIGSdb), Jolley and Maiden (2010)).

Based on the sequences of the four loci, the isolates were assigned to sequence types. Sequence types newly identified in this study were then attributed to clonal complexes using the clustering approach implemented in the program goeBURST v. 1.2.1 (http://goeburst.phyloviz.net, Francisco et al. (2009)). Sequence types belong to the same clonal complex when they share at least three identical alleles (out of four). To place our 160 Swiss *M. plutonius* isolates in the global phylogenetic context, we used the PHYLOViZ 2.0 program (Francisco et al., 2012, Nascimento et al., 2017) to construct a goeBURST tree including our samples and the 385 isolates available from the PubMLST database (http://pubmlst.org/mplutonius, as per June 2, 2022).

Further analyses to confirm the phylogenetic relationship were conducted on concatenated sequences of each sequence type (1904 bp). The allele sequences aligned using MAFFT (Kuraku et al., 2013) were downloaded from the PubMLST database in fasta format. The phylogenetic history was inferred with the maximum likelihood method (500 replications) and the Tamura-Nei model (Tamura and Nei, 1993) as implemented in MEGA11 (Tamura et al., 2011).

### 2.3 *mtxA* screening

To identify the presence of the putative virulence factor Melissotoxin A coded by *mtxA* (Djukic et al., 2018, Grossar et al., 2020), we screened all 160 isolates using PCR. As *mtxA* is located on pMP19, a plasmid that is prone to be lost by repeated sub-cultivation *in vitro* (Djukic et al., 2018, Grossar et al., 2020, Nakamura et al., 2020), we used DNA extracts of single step *M. plutonius* cultures of every isolate. Each isolate was screened for the corresponding gene with specific primers (*mtxA*, 897 bp; tox_MEPL_for: 5′-GCTCAAGCAGCAACTTTTACG-3′ and tox_MEPL_rev: 5′-TTCCCCTGGTATTACTTGTAGATG-3′). These primers were run in a conventional PCR reaction (initially 95 °C for 5 min, followed by 40 cycles of 95 °C for 15 s, 59 °C for 15 s, 72° C for 20 s, and final extension at 72 °C for 2 min), using KAPA2G Fast DNA Polymerase (KAPA Biosystems, USA). The total reaction volume was 25 µl consisting of 13 µl Kapa (2x) mix, 1 µl tox_MEPL_for at 10 µM, 1 µl tox_MEPL_rev at 10 µM, 8 µl water and 2 µl template DNA extract (10 ng DNA) in elution buffer (5 mM Tris/HCl, pH 8.5). The PCR-products were loaded on agarose gels, as described above for MLST.

### 2.4 **Isolate** mapping

Each *M. plutonius* sample (isolate) was mapped based on the zip code of the collection location using Quantum GIS (QGIS, version 1.8.0-Lisboa; http://www2.qgis.org/en/site, GNU General Public License; background maps and geographical information were obtained from www.toposhop.admin.ch).

### 2.5 Statistical **analysis**

To test whether the presence of *mtxA* was associated with the sequence type, clonal complex, or period of sampling, we calculated χ^2^ for maximum likelihood contingency tables (SPSS v. 26 (IBM, USA)). To determine whether the presence of *mtxA* promoted the spread of sequence types that have many isolates carrying this gene, we performed a Spearman rank correlation analysis between the frequency of each sequence type among all *M. plutonius* samples and the frequency of isolates harboring *mtxA* within each sequence type, among the samples of 2006–2007, of 2013 and for both sampling periods.

## 3 Results

### 3.1 Sequence types of *M. plutonius* from Switzerland

**Fig. 1:**
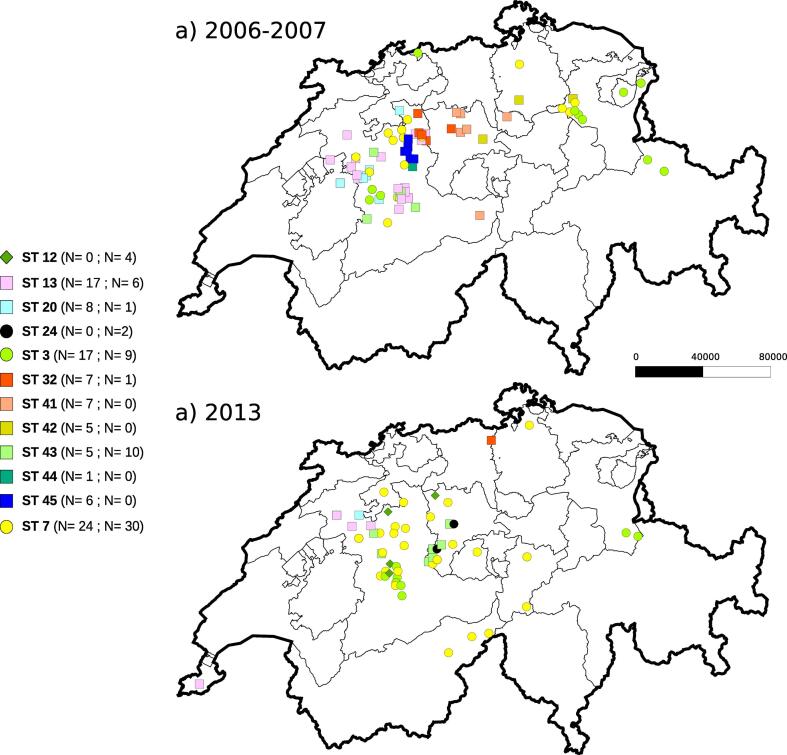
Distribution of sequence types of the 160 *M. plutonius* isolates collected in Switzerland over two sampling periods: a) 2006–2007 (N = 97) and b) 2013 (N = 63). The isolates belonged to 12 sequence types (in parentheses number of isolates of each sequence type detected in the first and second sampling, respectively), which grouped into three clonal complexes (circles = CC 3, diamonds = CC 12, and squares = CC 13).

**Fig. 2:**
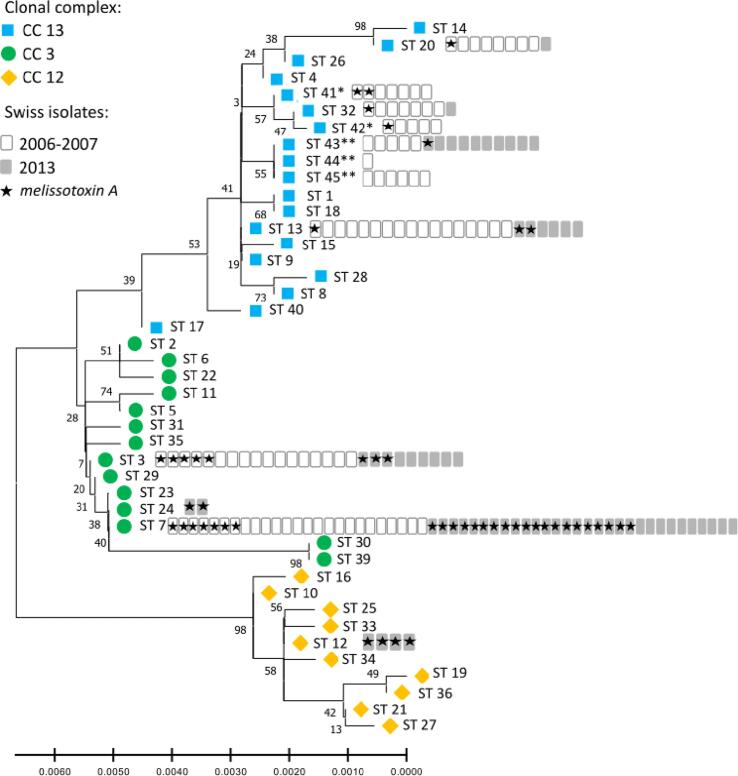
Phylogenetic tree of all known *M. plutonius* sequence types (ST) and clonal complex (CC), inferred with the maximum likelihood method and Tamura-Nei model, using the concatenated sequences of each sequence type. The tree with the highest log likelihood (-2949) is shown. The percentage of trees in which the associated taxa clustered together is shown next to the branches. * New sequence types identified in this study. ** New sequence types identified in this study with a new allele, galk12. *mtxA* was only screened in Swiss isolates collected in our study.

**Fig. 3:**
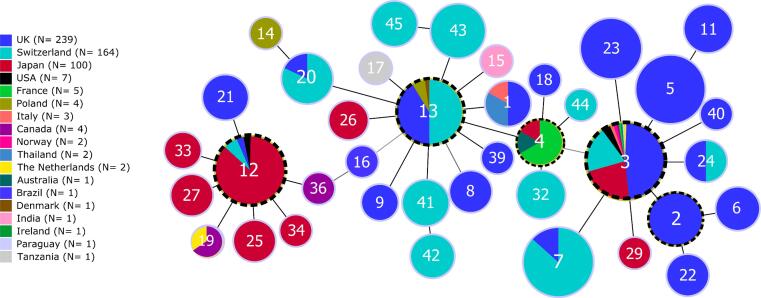
Clustering of *M. plutonius* sequence types based on sequence similarity, as per goeBURST analysis. The graph is based on MLST data from the current study and from the publicly available PubMLST database. Each circle represents a sequence type with its number of isolates in the center. Circle diameter correlates with sequence type frequency. Lines between circles link sequence types to their closest relatives. Black lines indicate single allelic changes between sequence types, whereas grey lines indicate differences at two loci. Circles with a dashed ring highlight putative founder sequence types. Colors indicate countries where the isolates of each sequence type were found (18 countries, including Switzerland). The three known clonal complexes are recognizable around the presumed founders ST 12, ST 13 and ST 3.

The 160 Swiss isolates of *M. plutonius* belonged to 12 sequence types (ST 3, ST 7, ST 12, ST 13, ST 20, ST 24, ST 32, ST 41, ST 42, ST 43, ST 44, and ST 45; Fig. 1 and Fig. 2) nested within three known clonal complexes (Fig. 3). The predominant clonal complex was CC 3 (52 % of the isolates analyzed), followed by CC 13 (46 %) and CC 12 (2 %; Fig. 1 and Fig. 2). ST 7 (34 % of the isolates analyzed) and ST 3 (16 %), both within CC 3, were the most frequently detected sequence types (Fig. 1 and Fig. 2). In four cases, two *M. plutonius* isolates were analyzed per sample, and both isolates belonged to the same sequence type. Of the 12 sequence types detected, five (ST 41 to ST 45) had never been identified previously in any of the 385 isolates listed in the PubMLST database. One previously unreported allele at locus galK distinguished three new sequence types (ST 43, ST 44 and ST 45). This new allele (galK 12) differs from the known allele galK 1 by a single nucleotide polymorphism (C > A) at position 257. The two other newly identified sequence types (ST 41 and ST 42) were based on new MLST profiles (new allele combinations), without novel sequence variation. Six sequence types were detected in both sampling periods (ST 3, ST 7, ST 13, ST 20, ST 32 and ST 43), comprising all the five sequence types that had been reported before. The rare sequence types ST 12 and ST 24 were not detected in isolates from 2006 to 2007, but occurred in two and four isolates from 2013, respectively (Fig. 2). Four of the five novel identified sequence types (ST 41, ST 42, ST 44 and ST 45) occurred only in the samples of the first period (2006–2007), but not in samples of 2013. ST 43 was the sole novel sequence type found in both sets of samples (Fig. 1 and Fig. 2).

### 3.2 Phylogenetic analysis of *M. plutonius* sequence types

As for the *M. plutonius* sequence typed in other countries, Swiss isolates clustered in three clonal complexes and their frequency distribution confirmed that sequence types occur at variable frequencies across countries and continents (Fig. 3). ST 3 was the most abundant sequence type identified worldwide, whereas it was the second most abundant sequence type in Swiss samples (Fig. 1 and Fig. 2). The maximum likelihood phylogenetic tree based on concatenated allele sequences of all 43 *M. plutonius* sequence types (Fig. 2) was highly congruent with the grouping inferred with goeBURST (Fig. 3). The goeBURST program recovered the three clonal complexes, the central position of the presumed founder sequence type in each clonal complex, and the phylogenetic relationships among most sequence types. The only major difference between the two analyses was the position of ST 39. This sequence type was grouped with CC 13 in the goeBURST clustering analysis (Fig. 3), because ST 39 is a double locus variant of ST 17 (differing at loci argE and gbpB). In contrast, ST 39 was assigned to CC 3 in the phylogenetic tree (Fig. 2), because ST 39 shares a single locus variant (galK) with many sequence types of CC 3 (Budge et al., 2014; ST 3, ST 5, ST 7, ST 23, and ST 24; de León-Door et al., 2018).

### 3.3 Distribution ***of mtxA***

Fifty out of 160 tested *M. plutonius* isolates (31 %) harbored *mtxA* (Fig. 2). The proportion of isolates carrying the *mtxA* gene increased from 19 % to 51 % between the 2006–2007 and the 2013 samples (Fig. 2). The proportion of isolates harboring *mtxA* within a sequence type was positively correlated with the frequency of this sequence type among all samples (samples of 2006–2007: Spearman’s-rho = 0.734, p = 0.007; samples of 2013: Spearman’s-rho = 0.923, p < 0.001; all samples: Spearman’s-rho = 0.684, p = 0.014). The sampling period was a significant factor explaining the occurrence of this putative virulence factor (maximum likelihood contingency table, χ^2^ = 5.40, dƒ= 1, p = 0.02). All four Swiss *M. plutonius* isolates belonging to CC 12 harbored *mtxA*, whereas 46 % of the isolates of CC 3 (N = 83) and only 11 % of the isolates of CC 13 (N = 74) harbored the gene. This frequency pattern leads to a significant association between *mtxA* occurrence and clonal complex (maximum likelihood contingency table, χ^2^ = 30.95, dƒ= 2, p < 0.001, N = 160). The same was true for sequence type (maximum likelihood contingency table, χ^2^ = 38.90, dƒ= 11, p < 0.001, N = 160; Fig. 2). In CC 3, *mtxA* was found in the two ST 24 isolates, in 51 % of the ST 7 isolates (N = 55) and in 31 % of the ST 3 isolates (N = 26). In CC 13 sequence types, *mtxA* frequency ranged from 0 % for ST 45 (N = 6) to 29 % for ST 41 (N = 7; Fig. 2). All four Swiss *M. plutonius* isolates belonging to ST 12 of CC 12 harbored *mtxA*.

## 4 Discussion

European foulbrood is a honey bee brood disease with a worldwide distribution. Although the disease has been known for a long time, we still lack information on the epidemiology of its etiological agent, *M. plutonius*. Here, we studied the genetic diversity, distribution, and population dynamics of *M. plutonius* in Switzerland. The Swiss honey bee populations serve as an excellent small-scale model with high apiary densities and therefore a high probability of disease transmission, which is attested by a high number of EFB cases. Using a multi-locus sequence typing (MLST) scheme, we identified 12 sequence types (of which five are novel) within 160 *M. plutonius* isolates sampled across Switzerland. The geographical distribution of the sequence types detected remained largely unchanged between the two sampling periods, but their prevalence, as well as that of *mtxA*, a gene coding for a putative virulence factor varied over time. The proportion of *mtxA* carrying isolates increased over time, in spite of the fact that the second sampling period occurring when the total number of cases reported in the country was decreasing.

The 12 sequence types detected among the 160 isolates belonged to the three known clonal complexes (CC; Budge et al., 2014). About half of the isolates were assigned to CC 3, the other half to CC 13, whereas CC 12 was rare and comprised only four 2013 samples. Sequence types ST 7 and ST 3, both belonging to clonal complex CC 3, were the most abundant sequence types in Switzerland, and are also very common worldwide (Budge et al., 2014, Haynes et al., 2013, Takamatsu et al., 2014). In England and Wales, ST 3 dominates, while ST 7 is less prevalent (Budge et al., 2014, Haynes et al., 2013). ST 13, which is the third most abundant *M. plutonius* type in Switzerland, has been detected in one sample from Denmark, three samples from Poland (Haynes et al., 2013) and 19 samples from England and Wales (Budge et al., 2014). The ubiquity of these sequence types suggests a large natural distribution range. In contrast, ST 12, the most abundant *M. plutonius* variant in Japan (Takamatsu et al., 2014), is only occasionally detected in other countries (de León-Door et al., 2018) and was absent from the 2006–2007 samples, but detected in four Swiss samples from 2013. ST 12 may thus have been recently introduced to Switzerland from one of the locations in which it was previously reported. Similarly, in England and Wales, one out of over 200 analyzed samples from 2011 to 2012 belonged to ST 12, consistent with a recent introduction (Budge et al., 2014). However, large-scale screenings of *M. plutonius* genetic diversity are rare, which limits our knowledge of the natural distribution range of sequence types and the role of honey bee related trade in explaining the observed biogeographic patterns (Mutinelli, 2011). Similarly, within country, human-mediated migration can contribute to spread sequence types. This latter effect is likely restricted in Switzerland, as only approximately 7 % of the beekeepers migrate their colonies to track nectar flows (Charrière et al., 2018).

Among the 12 sequence types detected in Switzerland, we identified five novel types (ST 41 to ST 45), to date unique to Swiss isolates. Three of these novel sequence types (ST 43, ST 44 and ST 45) included a yet unreported allele at locus galK (galK 12). The novel sequence types could either have been imported via trade of honey bees or of contaminated beekeeping material (Mutinelli, 2011), or have evolved in Switzerland. Evidence for a local evolution can be detected in the phylogenetic tree and goeBURST analysis (Fig. 2, Fig. 3). Indeed, ST 42 and ST 41 are closely related, and the first could have derived from the second. The same pattern was observed for ST 45 and ST 43. ST 44 is a single locus variant of ST 43 and ST 45. All three sequence types share the novel galK allele 12, which suggests that they are related by descent and thus of local origin. The goeBURST analysis also linked ST 44 to ST 4, because ST 44 is a single locus variant of ST 4, differing only by a single nucleotide polymorphism at the galK locus. ST 4 has not yet been detected in Switzerland, but it might have been introduced from France, Ireland or Japan, and remained undetected. Similarly, ST 32 is another sequence type found in Switzerland and closely related to ST 4. Finally, ST 4 is closely related to ST 3 and ST 13, both present in the country (Fig. 3). Overall, the phylogenetic pattern is largely consistent with local evolution of most novel sequence types, possibly following a transient introduction, e.g. of ST 4.

As there were numerous disease outbreaks in Swiss apiaries and the disease incidence was increasing rapidly at the time the 2006–2007 samples were collected, a local evolution of at least some of these novel types is in line with theoretical population genetic models. These models state that more mutations arise under expansion (*i.e.* increase in EFB cases), but either quickly die out or ‘surf’, *i.e.* become dominant (Miller, 2010). This scenario also helps account for why in 2013, when the incidence rates of EFB were already declining, only ST 43 appeared to have increased in prevalence, while all other novel sequence types (ST 41, ST 42, ST 44 and ST 45) were not detected again and likely had disappeared.

Other sequence types (ST 13, ST 20, ST 3 and ST 32), which had already been detected before our study, also decreased in prevalence in the second sampling period, despite a similar sampling effort. These results reveal high temporal dynamics in the genetic composition of the Swiss *M. plutonius* population. In contrast, the spatial dynamics appeared stable, as the same sequence types occured in the two sampling periods (Fig. 1). This spatial stability indicates that the pathogen had not spread over long distances in Switzerland, over seven years.

To investigate a potential link between *M. plutonius* virulence at the individual level (Grossar et al., 2020, Lewkowski and Erler, 2019) and spread (Alizon et al., 2009), we screened the *M. plutonius* variants for the presence of the recently discovered plasmid-encoded melissotoxin A toxin gene (*mtxA*) (Djukic et al., 2018). The toxin gene was found in all but two novel sequence types (ST 44 and ST 45) and was significantly associated with sequence type and clonal complex. Most interestingly, the gene was less frequently detected in isolates from 2006 to 2007, compared to isolates from 2013, which suggests that this gene could be an emerging virulence factor in the Swiss *M. plutonius* population.

In previous infection assays of honey bee larvae in the laboratory, we had detected significantly higher mortality when the larvae were infected with *M. plutonius* isolates harboring *mtxA*, compared to isolates lacking the toxin gene (Grossar et al., 2020). Because *mtxA* is carried by a plasmid, a mobile genetic element (Djukic et al., 2018), one can expect that *mtxA* is not present in all isolates of a given sequence type. Indeed, *mtxA* was found in varying proportions of the isolates of the detected sequence types (Fig. 2), suggesting this element can be acquired or lost by the pathogen. The conditions under which this plasmid is acquired or lost, and the dynamics of this phenomenon, are currently not understood.

In general, highly virulent pathogens are less likely to spread and persist in a population (Alizon et al., 2009), so that an inverse relationship between the percentage of *mtxA* carrying isolates and their prevalence in the field was expected. However, we observed the opposite trend, with the percentage of isolates carrying *mtxA* increasing between the first and the second sampling. This suggest that *mtxA* increased disease spread in Switzerland over a period of seven years, despite increased virulence. This pattern could be explained by an inverse relationship between individual and colony level virulence, due to earlier or more efficient hygienic behavior of adult honey bees directed against brood infected by highly virulent and damaging pathogens (Cremer et al., 2007, Page et al., 2016, Rauch et al., 2009). The relationship between individual and colony level virulence needs to be better understood to determine whether virulence-dependent control strategies can be applied (Grossar et al. 2020). In addition, *mtxA* is only one of several putative virulence factors (Djukic et al., 2018) and their respective roles in determining the spread of their carriers requires investigation.

To further complicate the relationship between virulence and spread of this honey bee pathogen, an interaction between the genome of each isolate and the presence of virulence-amplifying plasmids is likely as the presence of *mtxA* was significantly associated with sequence type and clonal complex. This is in line with results from genetic manipulation of Japanese *M. plutonius* isolates and infections of honey bee larvae *in vitro*. Nakamura et al. (2020) demonstrated that the plasmid containing *mtxA*, pMP19 (Djukic et al., 2018) increased the virulence of CC 3 isolates, but isolates of CC 13 were avirulent, irrespective of the presence of pMP19, and CC 12 isolates always caused high mortality rates, even in absence of pMP19. A further factor likely affecting the spread of isolates in the host population is the infections of colonies by multiple sequence types. Although in the four cases in which we typed two isolates per sample their sequence type was identical, infections by multiple sequence types were previously suspected (de León-Door et al., 2018, Djukic et al., 2018, Takamatsu et al., 2014). The interactions between sequence types via their effect on brood and colony health is likely influencing the ability of each sequence type to spread.

Despite a putative effect of *mtxA* gene on the spread of its carrier *M. plutonius* and an increase in the frequency of *mtxA* between the sampling periods, the total number of EFB cases in Switzerland did not further increase beyond 2010. The decrease in the number of cases was likely due to the stricter control measures implemented by veterinary authorities since 2010 ((FSVO), 2015) to counter the increased frequency of outbreaks experienced in the previous decade. From the mandatory destruction of symptomatic colonies and a control of the apiary at the end of the season before this date, later measures included the mandatory destruction of all colonies in an apiary if more than half of the colonies were symptomatic, and the monitoring of neighboring apiaries within a one kilometer radius after the report as well as at the next spring.

The data presented here for Swiss *M. plutonius* isolates, and comparison with data from other regions where EFB is currently becoming a sanitary problem, advance the knowledge of the epidemiology of EFB and offer a better understanding of the spread of genetic subtypes of *M. plutonius*. We found evidence that a high individual level virulence vectored by a mobile genetic element could favor the spread of *M. plutonius* isolates, but that increased control measures may have countered their propagation within the country. An improved understanding of the virulence factors within the genetically diverse *M. plutonius* is required to better mitigate future outbreaks. Combining large-scale monitoring of *M. plutonius* genotypes in data deficient regions with more detailed characterization of how *M. plutonius* impacts honey bees at the individual and colony level would be highly desirable, as this could provide key information to control this pathogen more efficiently and halt its spread worldwide.

## Declaration of Competing Interest

The authors declare that they have no known competing financial interests or personal relationships that could have appeared to influence the work reported in this paper.

## Data Availability

Data will be made available on request.
